# Postmortem metabolomics as a high-throughput cause-of-death screening tool for human death investigations

**DOI:** 10.1016/j.isci.2024.109794

**Published:** 2024-04-19

**Authors:** Liam J. Ward, Sara Kling, Gustav Engvall, Carl Söderberg, Fredrik C. Kugelberg, Henrik Green, Albert Elmsjö

**Affiliations:** 1 Department of Forensic Genetics and Forensic Toxicology, National Board of Forensic Medicine, 587 58 Linköping, Sweden; 2 Division of Clinical Chemistry and Pharmacology, Department of Biomedical and Clinical Sciences, Linköping University, 581 83 Linköping, Sweden; 3 Department of Forensic Medicine, National Board of Forensic Medicine, 587 58 Linköping, Sweden

**Keywords:** Death, Human, Biocomputational method, Metabolomics

## Abstract

Autopsy rates are declining globally, impacting cause-of-death (CoD) diagnoses and quality control. Postmortem metabolomics was evaluated for CoD screening using 4,282 human cases, encompassing CoD groups: acidosis, drug intoxication, hanging, ischemic heart disease (IHD), and pneumonia. Cases were split 3:1 into training and test sets. High-resolution mass spectrometry data from femoral blood were analyzed via orthogonal-partial least squares discriminant analysis (OPLS-DA) to discriminate CoD groups. OPLS-DA achieved an R2 = 0.52 and Q2 = 0.30, with true-positive prediction rates of 68% and 65% for training and test sets, respectively, across all groups. Specificity-optimized thresholds predicted 56% of test cases with a unique CoD, average 45% sensitivity, and average 96% specificity. Prediction accuracies varied: 98.7% for acidosis, 80.5% for drug intoxication, 81.6% for hanging, 73.1% for IHD, and 93.6% for pneumonia. This study demonstrates the potential of large-scale postmortem metabolomics for CoD screening, offering high specificity and enhancing throughput and decision-making in human death investigations.

## Highlights

• Postmortem metabolomics successfully discriminates five cause-of-death (CoD) groups
• Training and test sets resulted in true-positive prediction values of 68% and 65%
• Specificity optimization yielded 73%–99% prediction accuracies for the CoD groups
• Postmortem metabolomics shows potential for large-scale CoD screening

## Graphical abstract

**Figure:**
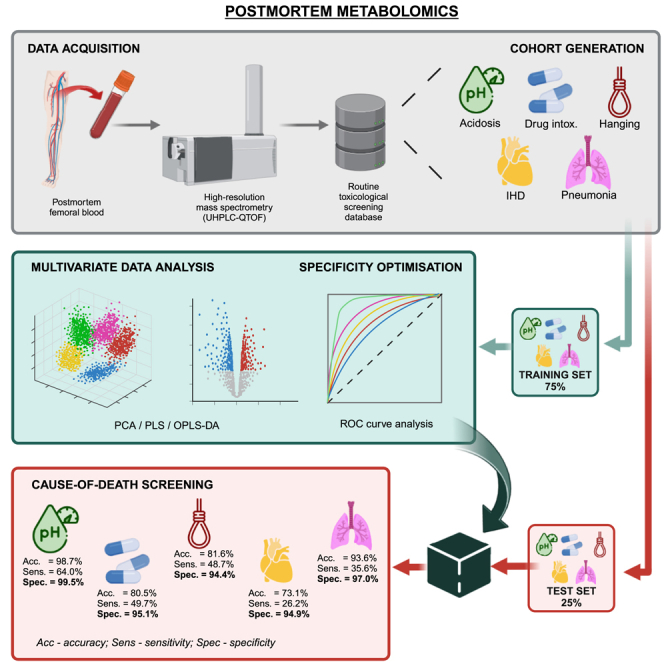

## Introduction

The primary objective of a death investigation, or postmortem autopsy, is to investigate and/or establish a cause-of-death (CoD). Globally, a general decrease in the frequency of postmortem examinations have occurred over the past decades, as observed across Western Europe, Australia, and the US.^1^^,^^2^^,^^3^^,^^4^ In Sweden, clinical autopsy rates have fallen from 40% to less than 5% between 1969 and 2016.^1^ Moreover, an analysis of over 1.8 million deaths across a 20-year period in Sweden concluded that a definite CoD, which is based on autopsy findings, was registered in only 12.6% of cases.^2^ Moreover, studies have found that in cases not autopsied, CoD diagnoses are more often unspecific and that CoD were missed in 30%–50% cases.^1^^,^^5^ It is essential to have accurate and complete CoD data for surveillance of health programs and policies, as this is a measure of how health conditions are evolving in terms of magnitude and distribution within populations.^6^ Moreover, accurate recording of mortality data is required for countries to progress toward the Sustainable Development Goals, with 7 goals and 17 indicators requiring cause-specific mortality data.^7^^,^^8^^,^^9^ An example of the effect of misclassifying CoD was reported during a study on colon and rectal cancer in California, USA, where reclassification of 82% of misclassified rectal cancer deaths to colon cancer resulted in a reduction of the overall 5-year survival rates in both colon and rectal cancers.^10^

CoD diagnosis by medical/forensic pathologists often relies on determining probabilities of cause-and-effect relationships through different methods, such as forecasting (observed cause → predicted effect) and back-casting (observed effect ← predicted cause), which often lack definitive criteria to establish validity.^11^ Therefore, diagnosing CoD is an acquired skill for pathologists and is reliant on experience, but also, having sufficient knowledge and data regarding the individual cases. Therefore, new technologies and methodologies are required to provide additional knowledge, increase throughput, and assist decision-making in death investigations.

When death occurs, together with the agonal period, a multitude of modifications and biochemical alterations occur within the body that may be investigated using metabolomics.^12^^,^^13^ Postmortem metabolomics has been investigated as a potential tool in aiding the determination of CoD. Previously, the utility of postmortem metabolomics has been demonstrated by proposing metabolic fingerprints, from femoral blood samples, for various CoD including pneumonia,^14^ oxycodone intoxications,^15^ and insulin intoxications.^16^ Postmortem metabolomics has also identified metabolic fingerprints for hypothermia, using vitreous humor samples,^17^ as well as differentiating central-nervous-system-related deaths based on the cerebrospinal fluid metabolome.^18^ Postmortem redistribution of exogenous substances has also been investigated using postmortem metabolomics.^19^ Recently, a multi-omics strategy has been employed to investigate postmortem interval (PMI) using bone samples from deceased humans; interestingly, here it was concluded that biomolecules predominantly from the metabolomics block showed best potential for PMI estimation, specifically for those with short- to medium-term PMI.^20^ Elsewhere, postmortem metabolomics has been utilized for the determination of PMI in numerous animal models.^21^^,^^22^^,^^23^^,^^24^

Postmortem metabolomics can reflect biochemical changes indicative of the agonal phase, CoD, and postmortem alterations. In Sweden, all forensic autopsies are subjected to a medicines and drugs screening using high-resolution mass spectrometry. Therefore, by reanalyzing these data, the potential to utilize metabolomics to screen various CoDs is possible, while maintaining a low cost and high throughput. This may provide additional support to medical/forensic pathologists during CoD diagnoses. Consequently, this can enhance the accuracy of CoD statistics, facilitating policy- and decision-makers to develop more refined surveillance programs and address public health challenges more precisely.

The aim of this investigation is to test the ability of postmortem metabolomics to be used as a screening tool on a large scale (>4,000 human cases) to differentiate five CoD groups: acidosis, drug intoxication, hanging, ischemic heart disease (IHD), and pneumonia. The hypothesis for this investigation is that by using postmortem metabolomics to train a screening model, we will be able to differentiate cases from an independent test group and screen them correctly into the five CoD groups.

## Results

### Study population characteristics

Study population characteristics for each included group are presented in Table 1, with specific CoD coding included in each group provided in Table S1. Expectedly, with distinctly different modes of death, significant differences in age, body mass index (BMI), PMI, and sex distribution across all five CoD groups were apparent. Those cases that died of drug intoxication and hanging were younger than those that died of acidosis, IHD, and pneumonia. The sex distribution across all groups were skewed toward males, with IHD and hanging groups having the highest percentage of males compared to females. Both drug intoxication and IHD groups presented with a median BMI >25, indicating a higher percentage of overweight cases in comparison to the other groups. For PMI both drug intoxication and hanging groups presented with median lower intervals compared to the other groups.

**Table 1:** Study population case characteristics per cause-of-death group included

	Acidosis, *n* = 100	Drug intox., *n* = 1385	Hanging, *n* = 1200	IHD, *n* = 1362	Pneumonia, *n* = 235	*p* value
Age, years	62 (55–68)	39 (29–54)	45 (30–60)	65 (57–73)	63 (55–71)	<2.2E-16
Sex, F/M %	30/70	35/65	22/78	20/80	33/67	<2.2E-16
BMI, kg/m^2^	22.8 (19.4–27.1)	27 (23.11–31.2)	23.9 (21.4–27)	26.4 (23.4–30.4)	23 (18.2–27.5)	<2.2E-16
PMI, days	6 (4–8)	5 (4–7)	5 (3–7)	6 (4–8)	6 (4–8)	1.2E-15

See also Table S1 for a full list of included CoD diagnoses per group.

Data presented as median (quartile range, Q1–Q3) were appropriate. Statistical analyses performed in accordance to data type and data distribution; chi-squared analysis for sex, Kruskal-Wallis non-parametric test for age, BMI, and PMI.

BMI, body mass index; F, female; IHD, ischemic heart disease; intox., intoxication; M, male; PMI, postmortem interval.

Principal-component analysis (PCA) for each CoD group identified less than 3% of cases as potential outliers (*n* = 119), which were excluded from model building (zero cases for acidosis, 38 for drug intoxication, 33 for hanging, 44 for IHD, and 4 for pneumonia).

### Control for features associated with case characteristics

Potential biases in the metabolome related to case characteristics were controlled for via a series of partial least squares (PLS) analyses or PLS-discriminant analyses (PLS-DA) models for each CoD group. Additionally, as data were collected from a total of 641 analytical runs from the four-year inclusion period, internal standard data were extracted from the quality control samples included in every analytical run. The variation in peak area for each internal standard was 25.0% for amphetamine-D8, 23.6% for diazepam-D5, and 21.1% for mianserin-D3 (Table S2). Therefore, to address the potential batch effects in the data, PLS models were also generated to control for sample run date. PLS/PLS-DA loading plots for age, sex, BMI, PMI, and sample run date for each CoD group are presented in Figure S1, and exclusion thresholds were set for positive and negative correlation values of p(corr) <−0.5 or >0.5. Sample run date produced PLS models for all five CoD groups, and there were 85 features surpassing the threshold in three of more CoD groups that were excluded. Age produced PLS models for all CoD groups, except acidosis, with 39 features surpassing the threshold in three or more CoD groups. Sex only produced PLS-DA models for drug intoxication and IHD groups; therefore, as sex was unable to explain the variance in metabolite features in the majority of CoD groups, no features were excluded. BMI produced PLS models for all CoD groups, and 49 features were excluded. PMI produced PLS model in four CoD groups, with acidosis not generating a model, and here 110 features were excluded. In total 269 unique features were excluded after controlling for potential confounders.

### Cause-of-death group discrimination and model validation

**Figure 1:**
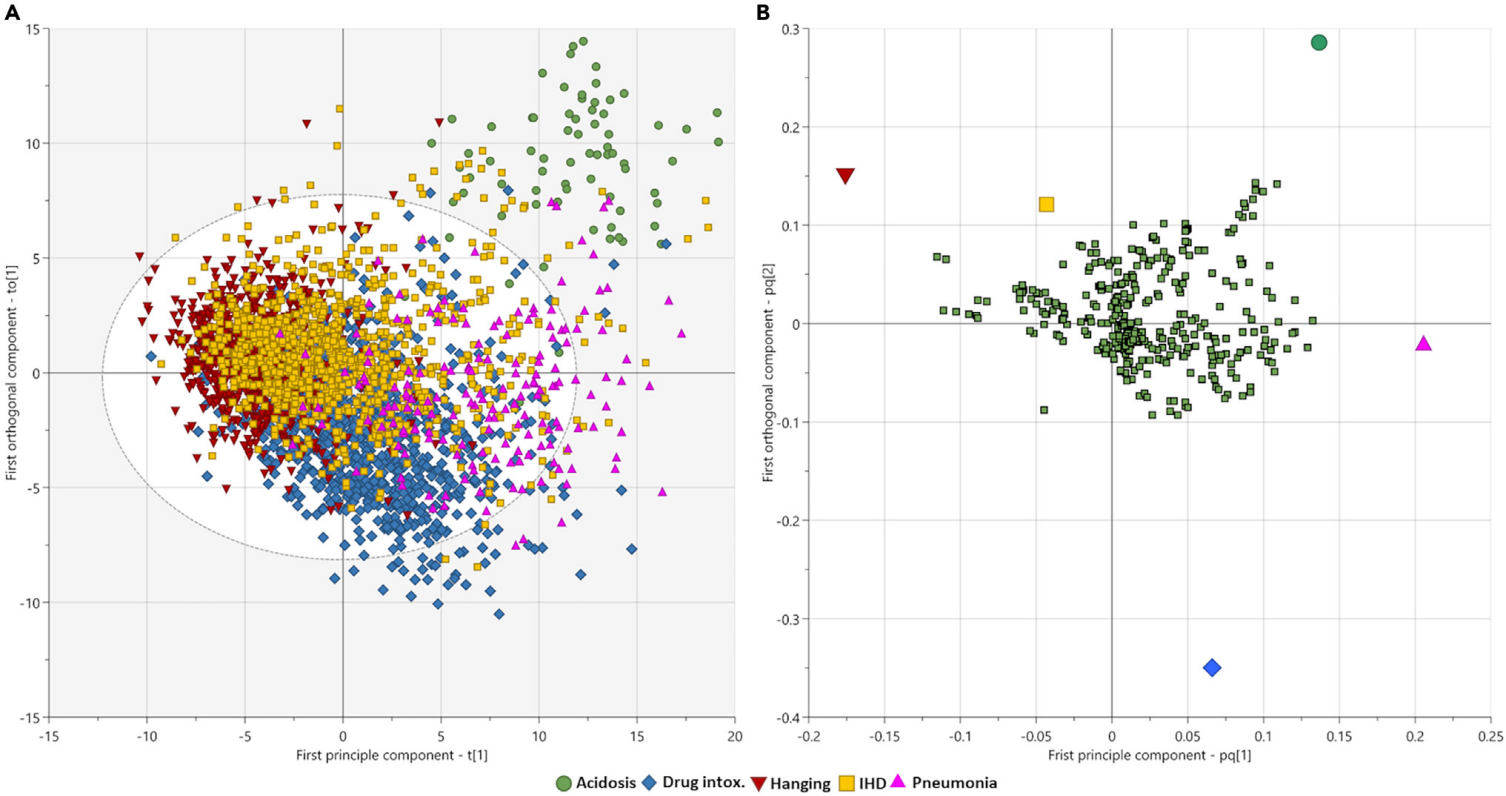
Cause-of-death group discrimination and model validation Multivariate modeling for discrimination of cause-of-death (CoD) groups using the training dataset (*n* = 3212). Orthogonal partial least squares-discriminant analysis (OPLS-DA) was performed sequentially in two iterations. First including all 1,052 metabolite features and then secondly, after exclusion of features below a variable influence on projection (VIP) score <1.0 with plots presented here with an R2 = 0.54 and a Q2 = 0.30. (A) OPLS-DA score plot showing distribution of the five CoD groups; acidosis (green circles), drug intoxication (intox.; blue diamonds), hanging (red inverted triangles), ischemic heart disease (IHD; yellow squares), and pneumonia (pink triangles). (B) Loading plot of metabolite features (green squares) with a VIP >1.0, relative to variance of the CoD groups.

The remaining 1,052 metabolite features were then used for orthogonal PLS-DA (OPLS-DA) modeling of the training set samples to distinguish the five CoD groups. The initial OPLS-DA model was generated with an R2 = 0.33 and a Q2 = 0.21. After exclusion of features below the variable influence on projection (VIP) score of <1.0, a new OPLS-DA model was generated including 374 metabolite features with an R2 = 0.54 and a Q2 = 0.30. Score plots and loading plots for this OPLS-DA model can be found in Figure 1.

**Figure 2:**
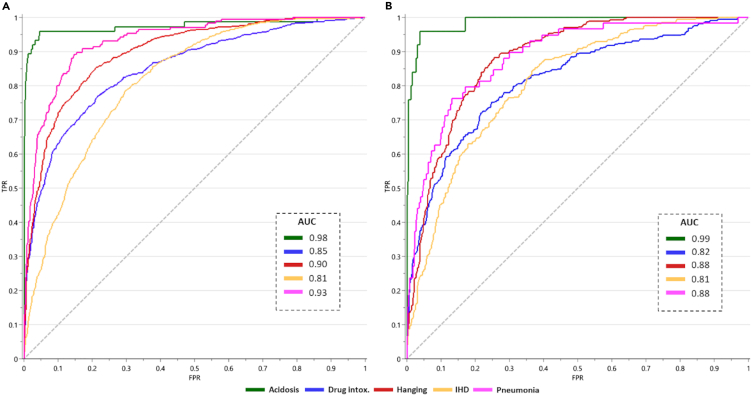
Model validation using an independent test set The cause-of-death (CoD) discriminant model was validated using prediction function for CoD, comparing the training and test datasets. (A) Receiver-operator-curves (ROC) for training set (*n* = 3212), with acidosis (green, AUC = 0.98), drug intoxication (blue, AUC = 0.85), hanging (red, AUC = 0.90), ischemic heart disease (IHD; yellow, AUC = 0.81), and pneumonia (pink, AUC = 0.93). (B) ROC for test set (*n* = 1070), with acidosis (green, AUC = 0.99), drug intoxication (blue, AUC = 0.82), hanging (red, AUC = 0.88), IHD (yellow, AUC = 0.81), and pneumonia (pink, AUC = 0.88).

In the evaluation of the training set OPLS-DA model, we utilized the class prediction function in SIMCA software to assign all samples from the training set, including those previously identified as outliers (*n* = 119), which were excluded during model building. Outliers were included here because they represent genuine cases with a diagnosed CoD; this inclusion aligns with the aim of the investigation of predicting any case, including outliers. This screening prediction was then compared to the true CoD diagnoses, which resulted in an overall true-positive prediction rate of 68% for the training set (Table 2). Validation of the multivariate model was conducted by performing the class prediction function on the independent test set of samples, representing 25% of each CoD group that have not been used in any of the model building. The test set class prediction was then compared to the true CoD diagnoses, which resulted in an overall true-positive prediction rate of 65% for the test set (Table 2). As both the training and test set predictions report similar sensitivities, 68% and 65%, respectively, it can be concluded that the models are not overfitted. Additionally, receiver-operator-curves (ROC) for both the training set and test set revealed similar area-under-curve (AUC) values for each CoD group, with the maximum deviation being a 5% AUC decrease for pneumonia CoD (Figure 2).

**Table 2:** Cause-of-death predictions for both the training and test set

*TRAINING SET*	True-positive	False-positive	*TEST SET*	True-positive	False-positive
Acidosis (*n* = 75)	45	**7**	Acidosis (*n* = 25)	14	4
Drug intox. (*n* = 1039)	744	274	Drug intox. (*n* = 346)	217	92
Hanging (*n* = 900)	666	327	Hanging (*n* = 300)	219	125
IHD (*n* = 1022)	696	409	IHD (*n* = 340)	236	144
Pneumonia (*n* = 176)	31	13	Pneumonia (*n* = 59)	9	10
***Overall (n = 3,212)***	***2,182 (68%)***	***1,030(32%)***	***Overall (n = 1,070)***	***695 (65%)***	***375(35%)***

Including the number predicted according to the model and number of correct predictions.

IHD, ischemic heart disease; intox., intoxication.

### Specificity optimization of class prediction

**Figure 3:**
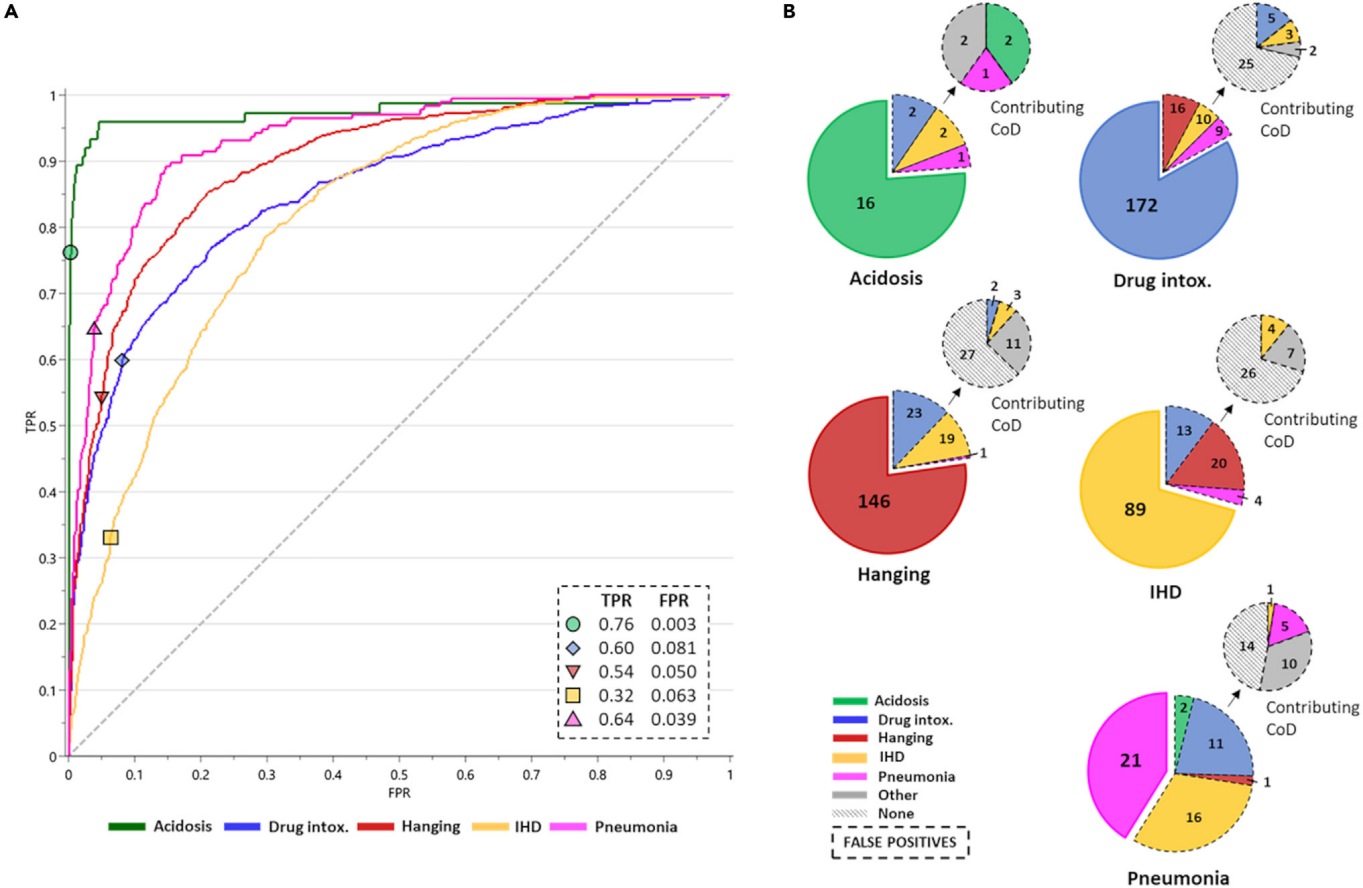
Cause-of-death screening using specificity-optimized thresholds (A) Screening thresholds calculated from receiver-operator-curve (ROC) for the training set (*n* = 3212), for each cause-of-death (CoD) group, within region >0.3 true-positive rate (TPR) and <0.1 false-positive rate (FPR), by calculating the steepest increment in each ROC curve. The calculated threshold for each CoD group relates to the following TPR/FPR values: 0.76/0.003 acidosis (green circle), 0.60/0.081 drug intoxication (blue diamond), 0.54/0.050 hanging (red inverted triangle), 0.32/0.63 IHD (yellow square), and 0.64/0.039 (pink triangle). (B) New CoD predictions in the independent test dataset (*n* = 1070) using specificity-optimized thresholds. False-positive predictions were investigated further using diagnosed primary and contributing CoD data. Only the specific CoD groups included in the study are indicated, including acidosis (green), drug intoxication (blue), hanging (red), ischemic heart disease (IHD; yellow), and pneumonia (pink). Additionally, in the contributing CoD review other CoD (gray) and none other (black stripes) are presented. The numbers in the figures represent the number of cases. See also Figure S2.

The default class prediction function generates prediction based on equal sensitivity and specificity. Thus, to evaluate the specificity further, we calculated prediction thresholds from the ROC for each of the CoD groups in the training set. The individual CoD predication thresholds were calculated from the steepest segments of the ROC curves, within the predetermined region of >0.3 true-positive rate (TPR) and <0.1 false-positive rate (FPR) (Figure 3A). Using the ROC thresholds for class prediction for the test set resulted in an increase in specificity. The test set was then predicted again using the ROC thresholds that resulted in 56% (*n* = 594) of cases being predicted with a unique CoD, 38% (*n* = 411) of cases unable to be predicted to a CoD group, and 6% (*n* = 65) of cases predicted in two CoD groups simultaneously. No cases were predicted in three or more CoD groups. A confusion matrix for true cases versus predicted cases using the test set is presented in Figure S2. The summary of the predictions for each CoD group including positive/negative prediction values, sensitivity, specificity, and accuracy is presented in Table 3.

**Table 3:** Test set screening prediction using calculated receiver-operator-curve thresholds

	PPV	NPV	Sensitivity	Specificity	Accuracy
Acidosis	76.2	99.1	64.0	99.5	98.7
Drug intox.	83.1	79.8	49.7	95.1	80.5
Hanging	77.2	82.5	48.7	94.4	81.6
IHD	70.6	73.4	26.2	94.9	73.1
Pneumonia	41.2	96.3	35.6	97.0	93.6

Data presents as %. IHD, ischemic heart disease; intox., intoxication; NPV, negative predictive value; PPV, positive predictive value.

The ROC-threshold prediction resulted in fewer predictions and true-positives for the CoD groups with larger populations (drug intoxication, hanging, and IHD) and more prediction and true-positives for the CoD groups with smaller population (acidosis and pneumonia). The overall sensitivity and specificity using the ROC thresholds were 42% and 86%. As a reference, the ROC thresholds were also applied to the prediction of the training set, which resulted in a similar sensitivity and specificity of 46% and 87%, respectively.

### False-positive investigation

False-positives were predicted in all of the CoD groups, with drug intoxication and IHD cases being falsely predicted in all other CoD groups; hanging cases falsely predicted as drug intoxication, IHD, and pneumonia; and acidosis cases only falsely predicted as pneumonia (Figure 3B). For all false-positive predictions, the contributing CoD diagnoses were reviewed, as these were not part of the inclusion/exclusion criteria during the study setup, to determine the frequency of matching contributing CoD. A contributing CoD is defined as a cause that is not deemed to be included in the primary chain of morbid events (see STAR Methods description for an example) but evident during a postmortem death investigation. For acidosis, 40% of false-positive cases had a matching contributing CoD, for drug intoxications 11% of cases had a matching contributing CoD, for hanging no cases had a matching contributing CoD, for IHD 11% of cases had a matching contributing CoD, and for pneumonia 17% cases had a matching contributing CoD (Figure 3B). Overall, matching contributing CoD diagnoses to the primary CoD screening were present in most groups, with the exception of hanging with no matching contributing CoD diagnoses.

### Metabolite identification and hierarchical clustering

**Figure 4:**
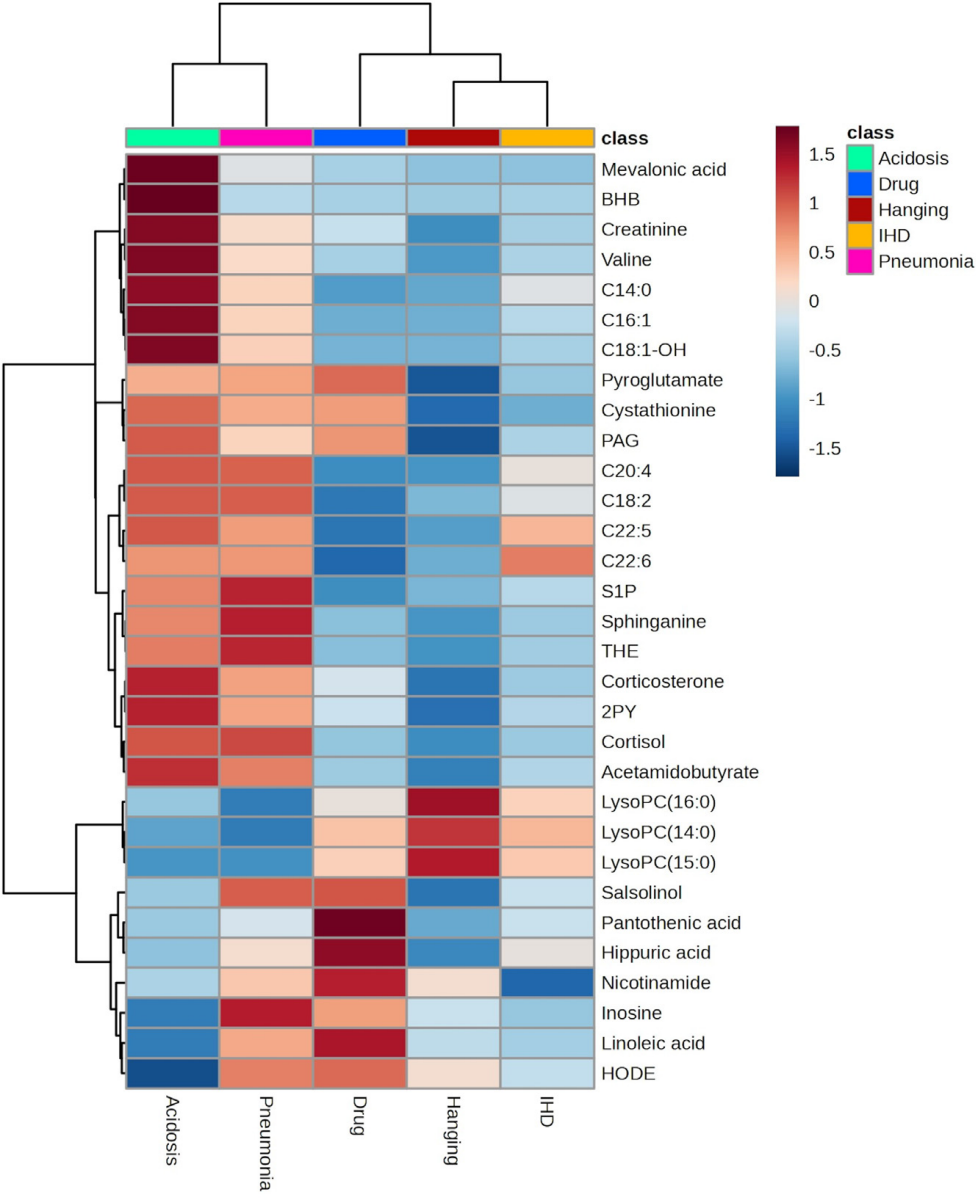
Hierarchical clustering of identified metabolites Hierarchical clustering heatmap of discriminant metabolites that were identified and significantly different in relative abundance between cause-of-death (CoD) groups. Relative abundance data used were obtained from the complete dataset (*n* = 4282). Clustering was performed both on CoD groups and metabolites. Data expressed are log-transformed and scaled. Red color denotes a greater relative abundance scaling through to blue color denoting a lower relative abundance. CoD class groups colored for acidosis (green), drug intoxication (blue), hanging (red), ischemic heart disease (IHD), and pneumonia (pink). 2PY, methyl pyridine carboxamide; BHB, hydroxybutyric acid; C14:0, myristoyl carnitine; C16:1, hexadecenoyl carnitine; C18:1-OH, hydroxy-octadecenoyl carnitine; C18:2, linoleoyl carnitine; C20:4, arachidonoyl carnitine; C22:4, cervonyl carnitine; C22:5, clupanodonyl carnitine; HODE, hydroxyoctadecadienoic acid; LysoPC, lysophosphatidylcholine; PAG, phenylacetylglutamine; S1P, sphingosine-1-phosphate; THE – tetrahydrocortisone. See also Figure S3.

Chromatographic features that were included in the final OPLS-DA model were subjected to database matching using the mass-to-charge (m/z) ratio. From these, 43 features were matched corresponding to 33 metabolites. Univariate statistical analysis, using one-way analysis of variance (ANOVA), confirmed that 31 of the metabolites displayed significantly altered abundances between CoD groups. A hierarchical clustering heatmap of these 31 significant metabolites is presented in Figure 4. Herein, the acidosis group displays a majority of significant metabolites that were found with higher relative abundances than the other CoD groups, whereas in the hanging group the opposite was observed with more metabolites being found with lower relative abundances than other CoD groups. Interestingly, the CoD group clustering shows that the IHD and hanging groups are the most closely related in terms of identified metabolites, followed by drug intoxications, and acidosis and pneumonia groups are distinctly not clustered with the other CoD groups. Furthermore, a full heatmap clustering including all chromatographic features demonstrates the differential abundances of features discriminating the CoD groups and also shows the similar clustering pattern of CoD groups (Figure S3).

## Discussion

Recent advances in postmortem metabolomics have demonstrated the potential for aiding death investigations. Notably, in developed countries, there has been a significant decline in autopsy rates, resulting in fewer conclusive CoD diagnoses.^1^^,^^2^^,^^4^^,^^5^ Postmortem metabolomics, as a novel technique in CoD screening, has the potential to improve the quality and throughput of death investigations, with the potential to discriminate CoD attributable to natural or disease-related causes and external factors, like drug intoxication and hanging/suffocation. This could facilitate the prioritization of in-depth examination for cases that require heightened scrutiny. The addition of postmortem metabolomics into routine death investigations has the potential to increase the number and accuracy of CoD data, reducing missed or incorrect CoD, and allowing of better health policy and surveillance. To the best of our knowledge, the current study constitutes the largest human postmortem metabolomics investigation to date, analyzing 4282 human cases, focusing on using postmortem metabolomics to screen cases based on five major CoD groups. The results demonstrate the potential of postmortem metabolomics in CoD screening and provided insights into the metabolic profiles associated with different CoD groups.

The study population exhibited significant differences in case characteristics among the five CoD groups, including age, sex distribution, BMI, and PMI, which are likely to impact the metabolome. The impact of these variables on the postmortem metabolome remains largely unclear, with the exception of PMI; whereas, numerous studies on the living metabolome have explored these characteristics. Recent metabolomics research in the living has suggested potential aging markers such as kynurenine, indole-2-aldehyde and cyclo-(leu-pro),^25^ as well as various acylcarnitines.^26^^,^^27^ Sex-related metabolomic differences have been examined in a systematic review involving 32 studies, revealing an expected sex bias in creatinine, but a distinct lack of bias in acylcarnitines abundances.^28^ The impact of BMI on the metabolome is multifaceted, dependent on factors such as adiposity and muscle mass, with associations to amino acids, particularly branched-chain amino acids.^29^ Metabolomics is also being explored to help determine PMI. For the majority, PMI has been studied using various animal models in controlled environments,^21^^,^^22^^,^^23^^,^^24^ although recent investigations have been conducted using human bone^20^ and pericardial fluid.^30^ Controlling for these case characteristics is crucial to ensure reliable model performance, and in the current investigation statistical modeling was used to exclude features showing bias toward age, BMI, sex, and PMI, resulting in a more robust model.

Combining postmortem metabolomics and multivariate modeling in models has been shown to generate potential models for pneumonia^14^ and oxycodone intoxication,^15^ with independent validation resulting in sensitivity and specificity values 80%–86% and 76%–84%, respectively. In addition, postmortem metabolomics in a screening test was able to identify potentially hypoglycemia-related deaths in a large randomly selected cohort.^16^ The current study also supports the hypothesis that postmortem metabolomics can effectively discriminate CoD. Herein, using the independent test set of over 1,000 cases, the specificity-optimized model resulted in an average sensitivity of 45% and specificity of 96% for the five CoD groups. These results show that the postmortem metabolomics model can correctly screen and discriminate cases belonging to different CoD groups based on the femoral blood metabolome.

The IHD group had the lowest performance in the validation screening test, with 73% accuracy, likely due to the diverse and chronic nature of IHD conditions. Among the five CoD groups examined, metabolic changes associated with IHD at the time of death may be less distinct compared to the other groups. This is evident from IHD cases being falsely identified as positive in all other CoD groups. Within the IHD test group, some cases were falsely identified as hanging, drug intoxication, and pneumonia. The majority of false-positives were identified as hangings (54%) or drug intoxications (35%). Hanging false-positives in the IHD group were not unexpected, occlusion of cerebral blood vessels and vagal-mediated cardiovascular collapse are described pathophysiological mechanisms of deaths by hanging, alongside asphyxia.^31^ Therefore, overlapping alterations in the postmortem metabolome could be expected. Hierarchical clustering of identified metabolites revealed the IHD group’s close proximity to the hanging and drug intoxication groups, suggesting shared postmortem metabolomic patterns. Notably, differences in nicotinamide and long-chain acylcarnitine levels appear to distinguish the IHD group from hanging and drug intoxication groups. Nicotinamide, a form of vitamin B3, is a precursor to nicotinamide adenine dinucleotide (NAD^+^), a central metabolite for mitochondrial energy homeostasis.^32^ Reduced NAD^+^ levels are associated with aging, obesity, and hypertension, and manipulating NAD^+^ levels has been explored as a therapeutic target for cardiovascular disease. Notably, long-term nicotinamide intake lowered blood pressure and reduced the risk of cardiac mortality in a human cohort.^33^ In the current study, the IHD group exhibited the lowest nicotinamide levels, suggesting diminished NAD^+^ associated with cardiovascular diseases. Greater abundances of a number of long-chain acylcarnitines (C14, C18:2, C22:5, and C22:6) seem to distinguish the IHD group from the other clustered CoD groups hanging and drug intoxication. Elevated acylcarnitine levels may indicate disruptions in mitochondrial carnitine shuttle and beta-oxidation pathways.^34^ Previous metabolomics research has identified elevated levels of acylcarnitines, encompassing short-, medium-, and long-chain variants, in patients with cardiovascular disease.^35^^,^^36^^,^^37^ Notably, medium- and long-chain acylcarnitines have been proposed as predictors for cardiovascular mortality.^36^ Interestingly, a recent metabolomics study compared acute and chronic coronary artery disease, and medium- and long-chain acylcarnitines were significantly increased in the chronic group, with C18:2 being specifically correlated with coronary artery disease complexity.^38^ In the current study, the IHD group is a heterogeneous group of diverse cardiovascular conditions, making direct comparisons to previous studies challenging. Nevertheless, the consistent finding across studies that increased levels of acylcarnitines are indicative of cardiovascular disease lends further support to our observations. In particular, the alterations in relative abundances of nicotinamide and long-chain acylcarnitines suggest dysfunction in mitochondrial energy production as a distinguishable factor for the IHD group, particularly in discriminating it from the hanging and drug intoxication groups. Future studies evaluating metabolites related to mitochondrial energy production from both clinical and postmortem IHD cases would be beneficial.

The hanging group was predicted with an 82% accuracy in the validation screening test. Notably, lysophosphatidylcholines (lysoPCs), such as lysoPC(14:0), lysoPC(15:0), and lysoPC(16:0), were found at relatively increased abundances in the hanging group, being the most abundant of the identified metabolites in this group. While a direct link between death by hanging and lysoPC has yet to be established, a recent study utilizing a porcine model to identify early PMI markers found significant increases in lysoPC(O-16:0) and lysoPC(18:1) at time points between 6 and 24 h after death.^39^ However, due to the relative slow increases over time, the applicability of these lysoPC as markers for PMI was questioned. In addition, this recent study identified the same lysoPC found to discriminate the hanging group and observed significant increases at 18–24 h after death.^39^ The current study excluded features influenced by PMI bias, and although the current cases have much longer PMI (median 5–6 days, per group), distinct lysoPC differences were apparent between CoD groups. Therefore, postmortem lysoPC changes could be influenced by CoD, warranting consideration in studies exploring global PMI markers with metabolomics.

The drug intoxication group achieved an 81% accuracy in the validation screening test. Among the five CoD groups included, the drug intoxication group is likely the most heterogeneous, encompassing 12 individual CoD codes (Table S1) and a wide array of contributing drugs. Previous postmortem metabolomics investigations have been performed on oxycodone intoxications^15^ and insulin intoxications.^16^ Moreover, postmortem metabolomics has been utilized in the context of postmortem redistribution of morphine and methadone.^19^ In the previous oxycodone and insulin intoxication studies, 21 and 12 acylcarnitines, respectively, were observed at lower abundances in intoxication cases compared to controls.^15^^,^^16^ In the current study, acylcarnitines C14, C18:1-OH, and C18:2 were also identified, with the drug intoxication group showing the lowest relative abundance of these, as well as four other acylcarnitines, compared to the other CoD groups. Although the current study identified fewer acylcarnitines that discriminate the drug intoxication groups from the other CoD groups compared to previous studies, this could be attributable to the high degree of heterogeneity within this group. Lower expression of acylcarnitines were also observed in the hanging group. Therefore, this acylcarnitine profile could potentially indicate prolonged hypoxia resulting from intoxication-induced respiratory depression. Additional postmortem metabolomic studies aimed at intoxications resulting from different drug classes could help establish common and discriminant metabolic features.

The pneumonia and acidosis groups were predicted with a 94% and 99% accuracy, respectively, in the validation screening test. It is important to note that these groups had fewer cases compared to other CoD groups. However, the predictions for these groups were robust, with consistent performance across default and specificity-optimized models for training and test sets.

Postmortem metabolomics has already been shown to discriminate pneumonia cases from randomly selected controls, with a sensitivity of 86% and specificity of 84%.^14^ In the current prediction test, using the specificity-optimized model, a decreased sensitivity of 36% and increased specificity of 97% were found. Despite lower sensitivity, several metabolites exhibited similar abundance profiles in pneumonia cases. The current findings confirm the previous postmortem observations with increased abundances of tetrahydrocortisone, cortisol, and phenylacetylglutamine and decreased abundance of lysoPCs; lysoPC(14:0), lysoPC(15:0), and lysoPC(16:0) in pneumonia cases.^14^ Previously in a pilot study, serum level of lysoPCs in pneumonia patients were observed to be lower in patients who died, as compared to survivors, suggesting a prognostic value for mortality.^40^ Similarly, in a metabolomics investigation of COVID-19 patients, LysoPCs were found at decreased levels in patients who did not survive, including a lower level of LysoPC(16:0).^41^ The acidosis group also displayed a similar profile for these metabolites, although only two acidosis cases were falsely identified as pneumonia. The majority of false-positives (53%) originated from the IHD group, potentially due to shared systemic inflammatory responses and prolonged mortality. While the current models were aimed at finding metabolites that distinguished the CoD groups from one another, it may be of interest to further explore similarities in the metabolic fingerprints between pneumonia and IHD groups to discover any metabolites related to systemic inflammation.

In the prediction test, the acidosis group outperformed all other groups in terms of sensitivity and specificity, and even on review of the false-positive predictions 40% had acidosis diagnoses as a contributing CoD. The acidosis metabolic fingerprint displayed greater abundances of long-chain acylcarnitines (C14:0, C16:1, C18:1-OH), organic acids (hydroxybutyric acid and mevalonic acid), valine, and creatinine. Acylcarnitines, hydroxybutyric acid (BHB), and mevalonic acid are involved in mitochondrial energy metabolism, with acylcarnitines transporting fatty acids across the mitochondrial membrane, BHB being a ketone body produced by fatty acid breakdown, and mevalonic acid an intermediate in the synthesis of coenzyme Q used in the electron transport chain. These observations may be indicative of alternative energy metabolism pathways being activated, like in terms of diabetic ketoacidosis when glycolysis is impaired due to a lack of insulin. Interestingly, a metabolomics study investigating diabetic ketoacidosis, using living human serum samples, found that BHB, valine, and C14:0 were significantly increased in hyperglycemic patients with diabetic ketoacidosis, as compared to both hyperglycemic patients without diabetic ketoacidosis and non-diabetic obese controls,^42^ indicating that these alterations are specific to the acidosis state. These findings are consistent with the present study, where the acidosis group had markedly greater abundances of these three metabolites as compared to other CoD groups. Moreover, BHB is used as marker for acidosis in forensic investigations with testing, at our institution in Sweden, increasing substantially in recent years.^43^

In summary, the current investigation presents the results of the largest human postmortem metabolomics investigation to date and exemplifies the potential of postmortem metabolomics as a novel technique for CoD screening during death investigations. Through a comprehensive analysis of 3,212 forensic autopsy cases, we constructed a robust multivariate statistical model capable of discriminating between five major CoD groups based on postmortem metabolomics. Validation with the independent test set in 1,070 autopsy cases demonstrated the screening potential with high prediction accuracies, ranging from 73.1% to 98.7% across the CoD groups. Future investigations should aim to expand postmortem metabolomics research, encompassing additional cohorts and exploring variations within major CoD groups and new CoD groups. Moreover, strategies leveraging systems biology and artificial intelligence should be explored to develop complex decision models. In conclusion, this study established a foundation for large-scale postmortem metabolomics to serve as a valuable tool in death investigations, offering the potential to improve CoD screening, enhance mortality statistics, and advance quality control and surveillance in healthcare.

### Limitations of the study

All cases were collected and analyzed by the national laboratory at the National Board of Forensic Medicine in Sweden; therefore, the study population includes samples from the whole country across a four-year period. A limitation here may be that the results are not generalizable to other countries due to different population demographics, particularly given that case ethnicity and socioeconomic status are not included in the National Board of Forensic Medicine database. In addition, batch effects may have an impact on the data analyses as data were collated from a 4-year period. However, previous research supports the use of retrospective data obtained from robust and accurate methods with appropriate quality assurance, like the data collected here from accredited routine methods, for metabolomics investigations.^44^ Moreover, by controlling for sample run date in the workflow we have endeavored to reduce any batch effects present. Another key limitation is the reliability of the CoD diagnoses used as the reference for constructing and evaluating the models. These diagnoses could be affected by incomplete medical records and subjective assessments. In our study, we have to assume that the CoD diagnoses by forensic pathologists are accurate in order to determine sensitivity and specificity. However, it is noteworthy that in a clinical setting, although not directly comparable to a forensic setting, it has been shown that up to 15% of diagnoses can be incorrect.^45^ The CoD diagnoses are in line with Swedish forensic practice; therefore, CoD codes and names included in Table S1 may not be directly comparable with the practices in other countries, particularly in the case of the acidosis group, where the included CoD descriptions in this group may not be registered as a primary CoD in other countries and/or regions. The study was aimed at discriminating CoD groups based on the femoral blood metabolome alone, and therefore, the exclusion of features displaying bias toward age, sex, BMI, and PMI prior model building was a strength. An argument could be made that these background features could also be useful for screening purposes; for example, the sensitivity of IHD screening may have increased if we included features related to age and BMI, as IHD incidence is closely related to age and BMI. While such features could theoretically increase the sensitivity, the specificity would likely decrease with an increase in the number of false-positive screenings. More studies are warranted to investigate metabolic features associated with the case characteristics to determine if any would have specific diagnostic potential for inclusion into any screening models.

## STAR★Methods

### Key resources table

**Table:** 

REAGENT or RESOURCE	SOURCE	IDENTIFIER
**Biological Samples**		

Human postmortem femoral whole blood	The National Board of Forensic Medicine, Sweden.	N/A

**Chemicals, Peptides, and Recombinant Proteins**		

Amphetamine-D8	Cerilliant, TX, USA	A-018
Diazepam-D5	Cerilliant, TX, USA	D-902
Mianserin-D3	Cerilliant, TX, USA	M-901

**Software and Algorithms**		

MassHunter	Agilent Technologies Sweden AB, Sweden.	version 10.0; RRID: SCR_019081
SIMCA	Sartorius AG, Germany.	version 17; RRID: SCR_014688
R	R Foundation of Statistical Computing, Austria	version 4.1.2; RRID: SCR_001905
RStudio	Posit PBC, MA, USA	RRID: SCR_000432
Microsoft Excel	Microsoft, WA, USA	Microsoft Office 2019; RRID: SCR_016137
MetaboAnalyst	McGill University, Canada	version 5.0; RRID: SCR_015539
XCMS & CAMERA (customised code)	Ward et al.^16^	https://www.mdpi.com/2218-1989/13/1/5

**Other**		

Agilient 1290 Infinity LC	Agilent Technologies Sweden AB, Sweden	N/A
Agilent 6540 QTOF	Agilent Technologies Sweden AB, Sweden	N/A
Waters Acquity HSS T2 column	Waters Sverige AB, Sweden	SKU: 186003540

### Resource availability

#### Lead contact

Further information and requests for resources and reagents should be directed to and will be fulfilled by the lead contact, Liam J. Ward (liam.ward@liu.se).

#### Materials availability

This study did not generate new unique materials.

#### Data and code availability

• The raw mass spectrometry data reported in this study cannot be deposited in a public repository because of ethical restrictions on the reporting of data derived from routine investigation of deceased individuals. Pre-processed metabolomics data and summary data reported in this paper will be shared by the lead contact upon request.
• This paper does not report original code.
• Any additional information required to reanalyse the data reported in this paper is available from the lead contact upon request.

### Experimental model and study participant details

High-resolution mass spectrometry data from routine toxicological screening of postmortem femoral blood was retrospectively collected from 4282 human autopsy cases admitted to the National Board of Forensic Medicine, Sweden, between July 1017 and November 2020. Age, years (median, IQR) = 54, 36–67; sex, female *n* = 1137 (26%). The study analyzed data from a nationwide population, as routine toxicological screening is performed on all forensic autopsies in Sweden. However, specific data regarding ethnicity and socioeconomic status are not recorded in the National Board of Forensic Medicine database.

This study was approved by the Swedish Ethical Review Authority (Dnr 2019–04530). Informed consent was not applicable for this study.

### Method details

#### Study population and group selection

Autopsy cases admitted to the National Board of Forensic Medicine, Sweden, between July 2017 and November 2020 were available for inclusion in the study (*n* = 17,011). All forensic autopsies were conducted by registered forensic pathologists in accordance with Swedish forensic practice, including the nomenclature of cause-of-death (CoD) diagnoses. Cases were evaluated in accordance to predetermined inclusion and exclusion criteria. Inclusion criteria: primary cause-of-death (CoD) diagnosis belonging to acidosis, drug intoxication, hanging, ischaemic heart disease (IHD), or pneumonia; calculated BMI; postmortem interval from either a confirmed death date or calculated death date with <48-h deviation between “body found” and “last seen alive”; body condition of none or emerging putrefaction. Exclusion criteria: <18 years old; no available HRMS data, diagnoses from different CoD groups within the primary train of morbid events. An example of exclusion due to diagnoses from different CoD groups present in the primary train of morbid events could be: “1A – Bronchopneumonia ← 1B – Aspiration pneumonia ← 1C – drug intoxication”, as both pneumonia and drug intoxication are diagnosed, the autopsy case would be excluded. A full list of included diagnoses for each CoD group are included in Table S1. Those matching criteria were included for this investigation (*n* = 4282). For this study, five groups of cases were selected based on their primary CoD including acidosis (*n* = 100), drug intoxication (*n* = 1385), hanging (*n* = 1200), ischaemic heart disease (IHD; *n* = 1362) and pneumonia (*n* = 235).

#### Postmortem blood screening

Mass spectrometry data was collated retrospectively to the sample analyses, after study population inclusion. Standardised procedures for mass spectrometry analyses were carried out in accordance with previously described.^46^ Briefly, data have been derived from femoral blood samples that were collected from all postmortem cases and analyzed using an ultra-high-performance liquid chromatography-quadrupole time-of-flight mass spectrometry system (UHPLC-QTOF; Agilent 1290 Infinity LC with Agilent 6540 QTOF; Agilent Technologies Sweden AB, Sweden). The method used is routinely performed at the National Board of Forensic Medicine (Linköping, Sweden), and is an accredited method for the targeted analysis of medicine and drugs via UHPLC-QTOF in positive ionisation mode, however, data recorded and saved from the entire analytical run. Femoral whole blood samples were prepared by protein precipitation (acetonitrile:ethanol, 90:10), including the addition of three internal standards (amphetamine-*d*_*8*_, diazepam-*d*_*5*_, and mianserin-*d*_*3*_). Samples were then injected into the UHPLC-QTOF system. The chromatography separation is performed via a gradient elution on a C18 reverse-phase column (150 mm × 2.1 mm, 1.8 μM; Waters Acquity HSS T2 column, Waters Sverige AB, Sweden). MS data were collected in positive ionisation mode and the total acquisition time for each sample was 12 min. Quality control samples, blank drug-free bovine whole blood containing the three internal standards, were included in every analytical run at the beginning and end. Mass spectra were pre-processed using XCMS and CAMERA packages in R (v.4.1.2) for peak list generation and peak annotation, according to parameters detailed in full previously.^16^

#### Multivariate modeling to discriminate cause-of-death

Data were randomly divided into a training set, containing 75% of samples from each CoD group (*n* = 3212), and a test, containing the remaining 25% of samples (*n* = 1070). The training set was used for model building and optimisation of the model, and the test set was used to validate the model.

For the training set, unsupervised PCA models were built for each CoD group to investigate possible group outliers. Outliers were assessed by using both distance to model X (DModX) and Hotelling’s T2 values for moderate and severe outliers, respectively, and if a sample was identified by both outlier calculations it was excluded from the training set.

Potential confounders associated with case characteristics were controlled for by using several partial-least squares (PLS) models for each CoD group, each including an individual case characteristic as a Y-variable, including; age, BMI, PMI, and sample date. Additionally, PLS-discriminant analysis (DA) models were built to control for testing sex. X-variable exclusion criteria included a feature with p(corr) value <-0.05 or >0.05 in three or more of the CoD groups, indicating that the X-variable feature has a particular bias toward a specific case characteristic.

After outlier and confounder exclusions, the remaining samples and features were used in a supervised orthogonal PLS-DA (OPLS-DA) model to distinguish CoD groups. OPLS-DA is cross-validated, via a 7-fold partitioning of the data, where data is divided into seven sets and the model is built with the omission of one set, then rebuilt with the inclusion of this set and omission of the next set, so on and so forth until each of the seven set have been omitted. OPLS-DA results in R2 and Q2 values, which represent the goodness of fit and cross-validation prediction performance, respectively. This model was further optimised by excluding features from the with a variable influence on projection (VIP) value < 1.0 in the initial OPLS-DA model, then a subsequent OPLS-DA model was generated as the final model.

#### Model validation and cause-of-death screening

The test set, representing 25% of the overall cohort, was left independent to the training set that had been used to build the multivariate models discriminating the five CoD groups. Thus, this independent dataset could be utilised for prediction and model validation testing.

Validation was performed by using a screening model to predict CoD, using the class prediction function in SIMCA software, and comparing predictions to the correct CoD diagnoses provided by the forensic pathologists. This function predicts group classification based on the distribution and variance of metabolite features included in the final OPLS-DA model built from the training set. Initially, the test set was screened, with the setting that every case should be screened into a single CoD group. The sensitivity and specificity of the test det screening were then compared to that of the training set prediction as a reference.

Further testing of the potential of this CoD screening model was performed by selecting new thresholds for CoD group classification, with the aim to access how much of the training set could be correctly predicted with a high level of accuracy. For the training set, receiver-operator-curves (ROC) were plotted, and thresholds were calculated from the steepest region of each CoD group curve. The calculations were performed within the region where the ROC curve had >0.3 true positive rate (TPR) and <0.1 false positive rate (FPR). These calculated thresholds where then applied to a new CoD screening for the test set of cases, and by comparing to the correct CoD diagnoses, updated sensitivity and specificity values were determined for this ROC-screening model.

#### False positive investigation

The ROC-screening model was further evaluated by investigating false positive screening in the test set for each of the CoD groups. False-positives were investigated via a review of contributing CoD, using the same codes used to assign CoD group (Table S1), to determine the frequencies and any possible explanations for false-positive prediction. A contributing CoD is defined as a cause that is not deemed to be included in the primary chain of morbid events, but evident during a postmortem death investigation.

#### Metabolite identification and hierarchical clustering analyses

Metabolite identification was performed by matching the mass-to-charge (m/z) ratio to online public databases, the human metabolome database (HMDB) and/or METLIN. In addition, MetaboAnalyst 5.0 was used to perform hierarchical clustering analyses of discriminant and identified metabolite features resulting from the final OPLS-DA models. For the hierarchical clustering analyses, relative peak intensities previously normalised uses PQN were log-transformed and scaled (mean centered and divided by the standard deviation). Hierarchical clustering was performed on both the CoD group and metabolite/feature levels, using Euclidean distance measure and Ward clustering method, as presented with CoD group averages.

### Quantification and statistical analysis

To test for differences in case characteristics between the five CoD groups, Shapiro-Wilk test was used for normality, Kruskal-Wallis for age, BMI and PMI, and Chi-squared for sex distribution. To test for differences between identified metabolites between CoD groups, ANOVA was used. Univariate statistical tests were performed using R v4.1.2 (R Foundation for Statistical Computing, Austria) or Excel 2019 (Microsoft, WA, USA). For univariate statistical analyses, significance was defined as *p*-value <0.05.

For multivariate modeling, chromatographic features with a retention time <90 s and >660 s were excluded from analyses. The data were normalised using probabilistic quotient normalisation using a reference spectrum calculated from the median [M]+ peaks across the entire sample set. Multivariate statistical modeling was performed using SIMCA (v.17; Sartorius AG, Germany). Multivariate statistical modeling included PCA, PLS, PLS-DA, and OPLS-DA models. All data throughout the modeling were log-transformed and unit-variance (UV) scaled. PCA models were used to identify potential outliers in each of the CoD groups. Outliers were assessed by using both distance to model X (DModX) and Hotelling’s T2 values for moderate and severe outliers, respectively, and if a sample was identified by both outlier calculations, with a *p* < 0.05, it was excluded. PLS and PLS-DA analyses were used to exclude chromatographic features which showed specific correlation with a case characteristic (age, sex, BMI and PMI), please refer to Figure S1. X-variable exclusion criteria included a feature with p(corr) value <-0.05 or >0.05 in three or more of the CoD groups, indicating that the X-variable feature has a particular bias toward a specific case characteristic. OPLS-DA models were used to discriminate CoD groups from one another.
