# Supplementary material for: Postmortem metabolomics as a high-throughput cause-of-death screening tool for human death investigations
Source: iScience. 2024 Apr 19;27(5):109794. doi: 10.1016/j.isci.2024.109794 (PMC11070332; doi:10.1016/j.isci.2024.109794)
Supplement: Document S1. Figures S1–S3 and Tables S1 and S2 [file mmc1.pdf]

iScience, Volume *27*

Supplemental information

Postmortem metabolomics as a high-throughput cause-of-death screening tool for human death investigations

Liam J. Ward, Sara Kling, Gustav Engvall, Carl Söderberg, Fredrik C. Kugelberg, Henrik Green, and Albert Elmsjö

Table S1. Cause-of-death codes included in each study group, related to STAR Methods. Codes used by the Department of Forensic Medicine, National Board of Forensic Medicine, Sweden, with English-translated explanations and frequencies included in each cause-of-death group.

Code	Explanation	n
Acidosis group		*(100)*
250B	Diabetes with ketoacidosis	21
271W	Alcoholic ketoacidosis	1
276	Disturbances in fluid, electrolyte and acid-base balance	4
276C	Acidosis	74
Drug intoxication group		*(1385)*
962D	Insulin and/or antidiabetic agents	1
965A	Opiates and closely related narcotics	355
969A	Antidepressants	4
969H	Psychostimulants	85
969W	Other psychotropic agents	1
969X	Psychotropic agents, unspecified	2
970	Central nervous system stimulants	1
970W	Central nervous system stimulants, other specific agents	5
977	Intoxication with other/unspecified medicine and drugs	1
977K	Single-drug intoxication	433
977L	Poly-drug intoxication	494
995C	Non-specific adverse effects of pharmaceuticals, narcotic and substances	3
Hanging group		*(1200)*
994K	Hanging	1200
Ischaemic heart disease group		*(1362)*
410	Acute myocardial infarction	424
410A	Acute transmural myocardial infarction	2
410K	Hemopericardium as a complication of acute myocardial infarction	43
410X	Acute myocardial infarction, unspecified	8
411X	Acute ischaemic myocardial damage, diffuse	196
412	Old myocardial infarction	162
414	Ischaemic heart disease	1
414A	Coronary atherosclerosis	476
414W	Diffuse scarring of the heart muscles	50
Pneumonia group		*(235)*
480W	Pneumonia, COVID-19 related	7
480X	Viral pneumonia, unspecified	8
481	Lobar pneumonia	63
482B	Pneumonia caused by pseudomonas	1
484W	Pneumonia, interstitial inflammation	1
485	Bronchopneumonia	143
485X	Bronchopneumonia, unspecified	3
486	Pneumonia, lung/interstitial inflammation	1
486X	Pneumonia cause by non-specific microorganism	7
487	Pneumonia, influenza	1

Table S2. Summary of internal standard results across the inclusion period, related to STAR Methods. Variation in the internal standards from quality control samples, blank whole blood, included in analytical runs (n = 641) across the four-year inclusion period.

Internal Standards	Area Criteria	Mean Absolute Area	CV	rt (s)	Maximum rt deviation	m/z	Maximum ppm deviation
Amphetamine-D8	1.2E+06	2.2E+06	25.0%	217.5	±14.8	143.155	±5.7
Diazepam-D5	1.4E+06	2.7E+06	23.6%	566.7	±10.2	289.103	±4.2
Mianserin-D3	1.6E+06	3.0E+06	21.1%	412.0	±16.8	267.182	±4.0

July 2017–November 2020 (n = 641)

CV – coefficient of variance; m/z – mass-to-charge ratio; rt – retention time; ppm – parts per million

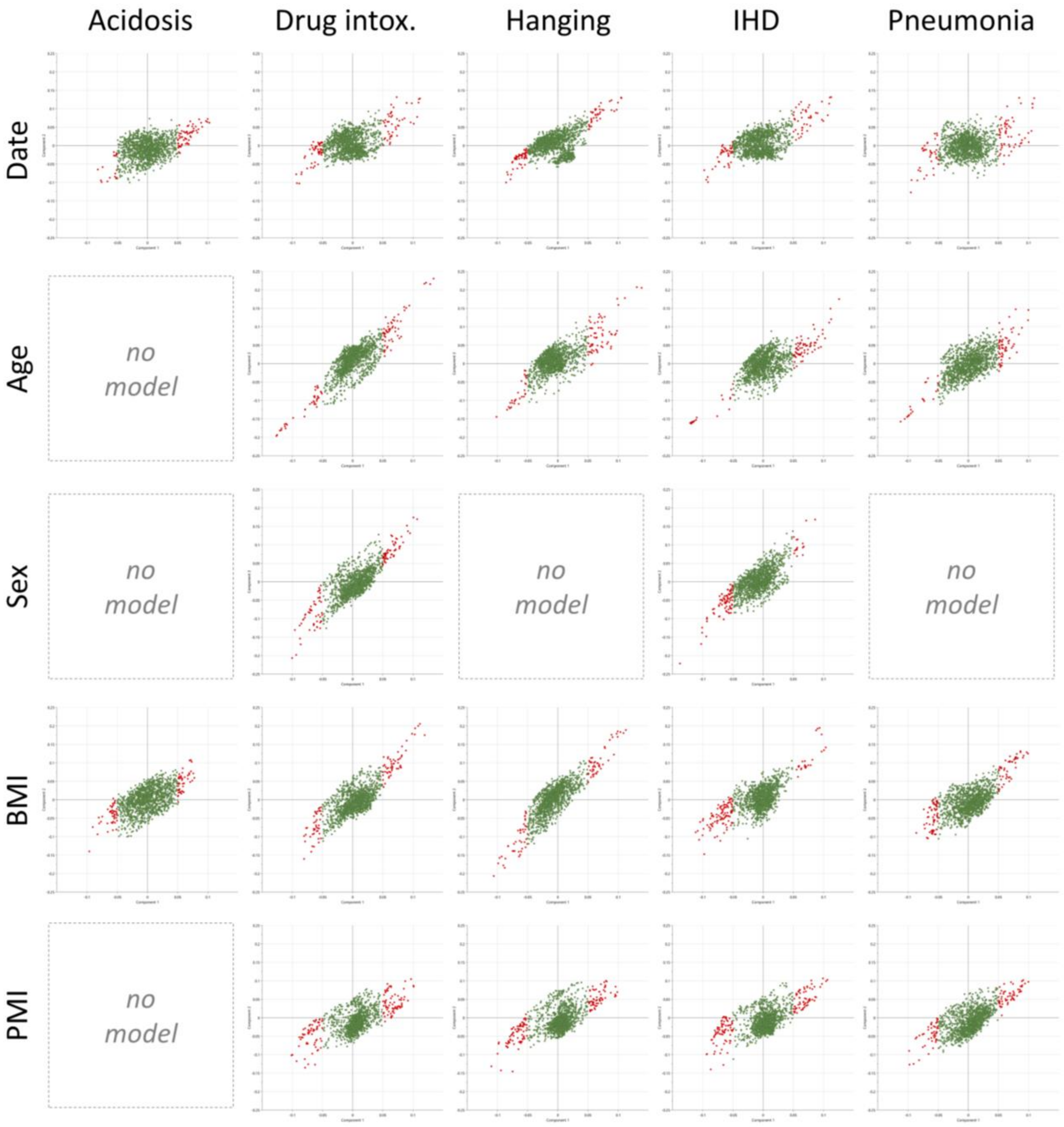

Figure S1. Controlling for features associated with case characteristics, related to Table 1. Multivariate modelling for potential confounding variables for each cause-of-death (CoD) groups. Several partial least squares (PLS)/PLS-discriminant analysis (PLS-DA; for sex variable) were generated for each CoD group using the training dataset, acidosis (n = 75), drug intox. (n = 1039), hanging (n = 900), IHD (n = 900), pneumonia (n = 176). Case characteristics or sample run date ("Date") were set as the descriptive Y-variable. Metabolite features, as X-variables, surpassing the threshold of p(corr) <-0.05 or p(corr) >0.05 are highlighted in red. If a highlighted feature surpasses the threshold in three of more CoD groups for each Y-variable, it is considered biased towards that Y-variable and excluded from further analyses. "no model" means that the variation in X-variables cannot be explained by the defined Y-variable. BMI – body mass index; IHD – ischaemic heart disease; PMI – postmortem interval.

TEST SET (n = 1070)		PREDICTED CASES					
		Acidosis	Drug intox.	Hanging	IHD	Pneumonia	*[Unclassified]*
TRUE CASES	Acidosis	16	0	0	0	2	7
	Drug intox.	2	172	23	13	11	125
	Hanging	0	16	146	20	1	117
	IHD	2	10	19	89	16	204
	Pneumonia	1	9	1	4	21	23

Figure S2. Cause-of-death prediction after specificity optimisation, related to Figure 3. Confusion matrix of test set prediction using the specificity-optimised receiver-operator-curve (ROC) threshold predictions.

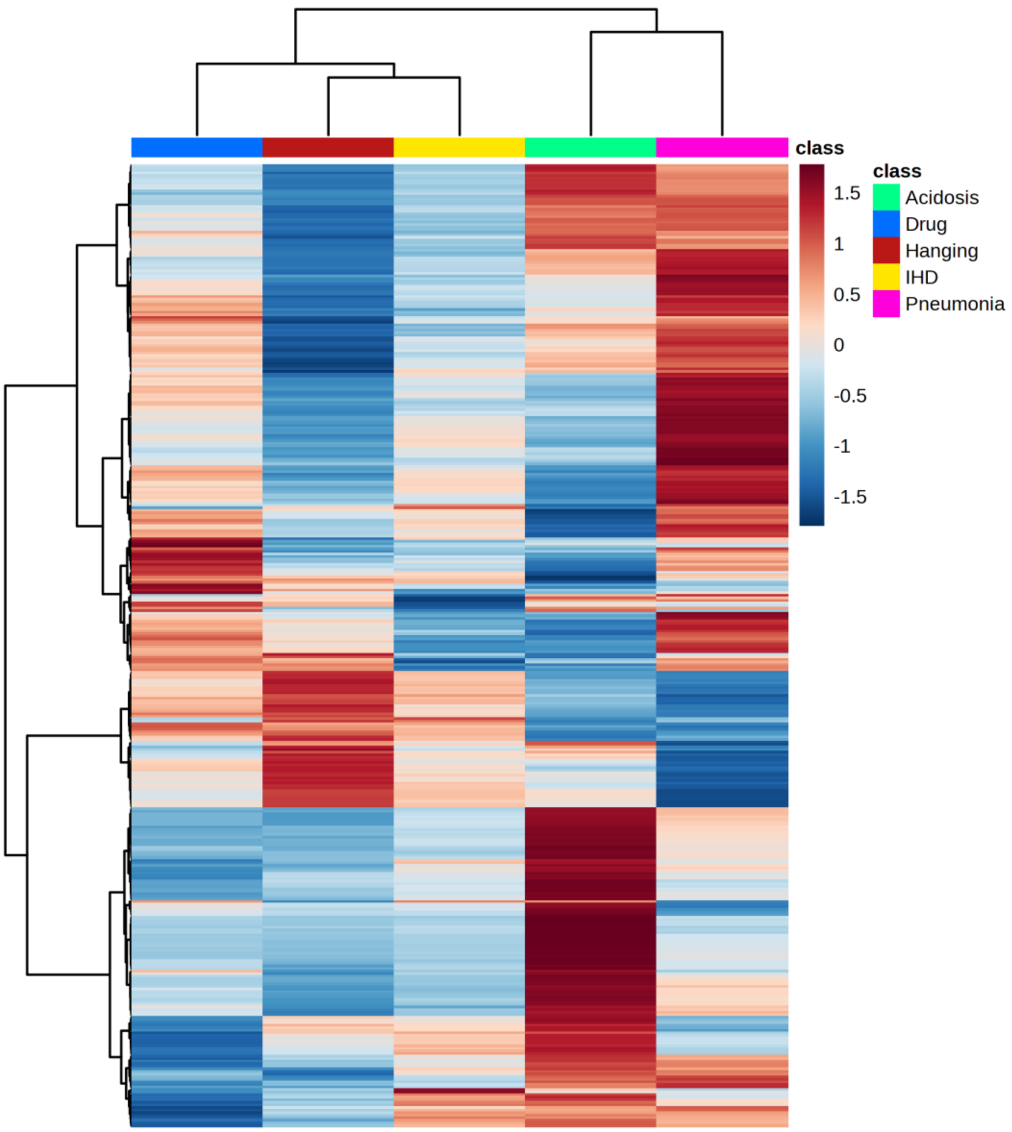

Figure S3. Hierarchical clustering of discriminant chromatographic features, related to Figure 4. Hierarchical clustering heatmap of all discriminant chromatographic features included in the final orthogonal partial least squares-discriminant analysis (OPLS-DA) used for prediction models. Clustering was performed both on cause-of-death (CoD) groups and chromatographic features. Data expressed are log-transformed and scaled. Red colour denotes a greater relative abundance scaling through to blue colour denoting a lower relative abundance. CoD class groups coloured for acidosis (green), drug intoxication (blue), hanging (red), ischaemic heart disease (IHD), and pneumonia (pink).
